# Outcome analysis for prediction of early and long-term survival in patients receiving intra-aortic balloon pumping after cardiac surgery

**DOI:** 10.1007/s11748-016-0679-3

**Published:** 2016-07-25

**Authors:** Hiroyuki Kamiya, Maximilian Schilling, Payam Akhyari, Arjang Ruhparwar, Klaus Kallenbach, Matthias Karck, Artur Lichtenberg

**Affiliations:** 1 Department of Cardiac Surgery, Asahikawa Medical University, Midorigaoka Higashi 2-1-1-1, Asahikawa, 078-8510 Japan; 2 Department of Cardiovascular Surgery, University of Duesseldorf, Moorenstrasse 5, 40225 Düesseldorf, Germany; 3 Department of Cardiac Surgery, University of Heidelberg, Im Neuenheimer Feld 110, 69120 Heidelberg, Germany

**Keywords:** Cardiac surgery, Low output syndrome, Intraaortic balloon pumping

## Abstract

**Background:**

Patients requiring an intra-aortic balloon pump (IABP) after cardiac surgery are critically ill and need a prolonged ICU stay. Considering limited health care resources, the early identification of patients with an extremely poor prognosis is important as a solid base for the decision whether further aggressive continuation or cessation of the therapy is recommendable.

**Methods:**

From 2001 to 2007, 552 patients with low-output syndrome after open-heart surgery and IABP implantation in OR or within 24 h thereafter on ICU were retrospectively analyzed.

**Results:**

The overall mortality at 30 and 180-day were 31 and 40 %, respectively. According to multivariate analyses, following factors were used to generate an IABP score: female gender, age ≥70 years, simultaneous coronary and valve surgery, aortic cross-clamp time >120 min., need of norepinephrin more than 0.4 µg kg^−1^ min^−1^, postoperative dialysis, and maximal serum creatinine kinase >3000 mg mL^−1^. The 30-day mortality continuously increased along the score (10.1 % for score = 0, *n* = 98; 11.8 % for score = 1, *n* = 144; 27.5 % for score = 2, *n* = 153; 40.4 % score = 3, *n* = 89; 65.2 % for score = 4, *n* = 46; 77.8 % for score = 5, *n* = 27) and reached 100 % for all patients with a score of 6 (*n* = 4).

**Conclusions:**

Prediction of 30 days mortality was possible with our scoring system based on multivariate analysis, and patients with scores of 4 or greater had remarkably worse early and late survival.

## Introduction

Intraaortic balloon pumping (IABP) is now the most commonly used mechanical assist device for postcardiotomy low output syndrome (LOS) [1]. There have been some efforts in the past to predict the prognosis in this patient cohort [2–6], including the introduction of scoring systems reported from two German large volume centers, the German Heart Center Berlin group [4] and Bad Oeyenhausen group [5]. The aim of previous efforts was focused on the identification of patients who would benefit from further aggressive therapy, e.g. with the implantation of an extracorporeal membrane oxygenation (ECMO) or a ventricle assist device (VAD). Therefore, those reports only considered acute parameters (serum lactate level, hemodynamics, mixed venous saturation, and adrenaline dose) obtained 1–6 h after surgery. In contrast, preoperative patient characteristics, intraoperative parameters and postoperative complications were not analyzed by those reports [4, 5].

However, it is sometimes very difficult to meet a decision for further therapy escalation immediately after surgery. For example, there are some patients with postcardiotomy LOS, who may initially be stabilized under IABP support and moderate inotropic doses, but who will remain at a remarkably high rate for renal failure, pneumonia or other complications, all rapidly leading to multi-organ failure. Moreover, even after successful early postoperative recovery and discharge from intensive and intermediate care unit the mid term results of these patients remain remarkably limited. For these patients, an alternative scoring system allowing the prediction of outcomes which can be applied not only to the very early postoperative period, but also to the postoperative course, may be helpful to identify patients who need further therapy escalation to maintain their midterm prognosis.

In recent years, the general cardiac surgical patient population has shifted towards an older cohort with increased complexity, and there are many patients in whom ECMO or VAD are absolutely, relatively or socially contraindicated. Previous reports were aimed only for further escalation of therapy to a point of ECMO or a VAD implantation. However, our health care resources have increasingly become under the pressure of financial limitations, excluding the strategy of general implantation of such devices in all patients in whom an IABP-support is not enough. An alternative scoring system taking patient characteristics, intraoperative parameter and postoperative complication into account may be also helpful to make a decision to therapy cessation, especially in a prolonged postoperative course.

The aims of this study were to clarify risk factors for and to establish a scoring system to predict early and long-term outcome in patients needing an IABP-support after cardiac surgery.

## Patients and methods

Between November 2001 and December 2007, 9243 patients underwent cardiac surgery and 649 patients (7.0 %) received an IABP due to perioperative cardiac low output syndrome (LOS) in our institute. Patient demographics, preoperative data, intraoperative procedures and postoperative data for the in-hospital course and the 180-day outcome were prospectively entered in an institutional data base. A retrospectively evaluation was performed for all 649 patients, additionally including a follow-up for the clinical outcome up to the time of the study. Of all patients, the use of IABP was started preoperatively in 47 patients, intraoperatively in 513 patients, postoperatively within the first 24 h in 39 patients, and in further 50 patients after the initial 24 postoperative hours. To focus on postcardiotomy LOS, patients who received an IABP preoperatively and late postoperatively after 24 h were excluded from further analysis. From the remaining 553 patients, one patient was excluded from further analysis because of lack of medical records. Thus, 552 patients (512 patients intraoperative IABP and 39 patients postoperative IABP within 24 h from the operation) were included in the analysis. All patients were operated on through the median sternotomy and cardioplegic cardiac arrest with Bretschneider-solution.

The indications for IABP implantation included the following: left atrial pressure or pulmonary artery wedge pressure increased by >18 mmHg, cardiac index decreased to <2 L min^−1^ m^−2^, and mean systolic arterial pressure <80 mmHg despite epinephrine support (>0.15 µg kg^−1^ min^−1^) for weaning form cardiopulmonary bypass intraoperatively or for new onset of postoperative low output syndrome. During IABP therapy, epinephrine was administrated as inotropic agent and norepinephrine was administrated for vasoconstriction. The indications for stopping IABP therapy included the following: stable hemodynamic with minimal epinephrine support (<0.05 µg kg^−1^ min^−1^) and no hemodynamic deterioration by reduction test of IABP to 1:3 modus. All patients received heparin infusion on the ICU with the partial thromboplastin time of 45–50 s.

For data acquisition, the documentation system of the *Heidelberger Verein für multizentrische Datenanalyse e. V.* (HVMD) was used, where about 1500 variables per case are entered in prospective manner according to their clinical course [7]. In this system, pulmonary hypertension was defined as mean pulmonary artery pressure more than 30 mmHg. Short-term mortality up to 180 days was also documented in HVMD-system. After approval of the institutional review board, follow-up was obtained through contact with local population administration office, home doctor, or with the patient/family directly. Completeness of follow-up was 97 %.

### Statistical analysis

Results are expressed as mean ± standard deviation. Statistical analysis was performed using Student’s *t*-test for continuous variables or *χ*^2^ tests (Fisher’s exact tests if *n* < 5) for categorical variables. Kaplan–Meier analysis was used to estimate late mortality between subjected groups. Logistic regression was also used for the multivariate analysis of risk factors for mortality. Receiver operating characteristics (ROC) curves were used to test discrimination power of our scoring system. A *p* value less than 0.05 was considered significant. All statistical analyses were performed using SPSS 16.0 software (SPSS Inc., Chicago, IL).

## Results

### Patient demographics and early outcome

Patient characteristics and intraoperative parameters are listed in Table 1. Early outcome including 30-day mortality and postoperative complications are listed in Table 2. The 30-day mortality was 30.8 % in the entire patient cohort. No patient received a ventricular assist device. Five patients received an extracorporeal membrane oxygenation and all of them died.

**Table 1:** Patients’ characteristics and intraoperative parameters

Preoperative factors	
Male (%)	372 (67.4 %)
Age (years)	66.7 ± 12.0
Body mass index (kg m^−2^)	27.0 ± 4.0
Diabetes mellitus (%)	96 (17.4 %)
Hyperlipidemia (%)	424 (76.8 %)
Hypertension (%)	481 (87.1 %)
Current smoker (%)	98 (17.8 %)
Pulmonary hypertension (%)	152 (27.5 %)
Peripheral artery disease (%)	104 (18.8 %)
COPD (%)	185 (33.5 %)
Renal insufficiency (%)	176 (31.9 %)
Renal insufficiency on dialysis (%)	15 (2.7 %)
Serum creatinine (mg/dL)	1.2 ± 0.8
Lever cirrhosis (%)	14 (2.5 %)
Poor LV function (%)	202 (36.6 %)
Emergency (%)	370 (67.0 %)
Re-do operation (%)	17 (5.3 %)
NYHA class IV, *n* (%)	309 (56.0 %)
Re-do, *n* (%)	77 (14.1 %)
Operation	
CABG	319 (57.8 %)
Valve	69 (12.5 %)
Aortic valve replacement	15 (21.7 %)
Mitral valve replacement	12 (17.4 %)
Mitral valve repair	6 (8.7 %)
Aortic and mitral valve replacement	10 (14.5 %)
Aortic valve replacement and mitral valve repair	4 (5.8 %)
Mitral valve replacement and tricuspid valve repair	5 (7.2 %)
Mitral valve repair and tricuspid valve repair	3 (4.3 %)
Aortic and mitral valve replacement and tricuspid valve repair	12 (17.4 %)
Aortic valve replacement and mitral and tricuspid valve repair	2 (2.9 %)
CABG + valve	118 (21.4 %)
Aortic valve replacement	45 (38.1 %)
Mitral valve replacement	12 (10.2 %)
Mitral valve repair	8 (6.8 %)
Aortic and mitral valve replacement	10 (8.5 %)
Aortic valve replacement and mitral valve repair	6 (5.1 %)
Mitral valve replacement and tricuspid valve repair	7 (5.9 %)
Mitral valve repair and tricuspid valve repair	6 (5.1 %)
Aortic and mitral valve replacement and tricuspid valve repair	15 (12.7 %)
Aortic valve replacement and mitral and tricuspid valve repair	3 (2.5 %)
HTx	22 (4.0 %)
Surgery on aortic arch	11 (2.0 %)
Miscellaneous	13 (2.1 %)
Intraoperative parameters	
Operation time (min)	308 ± 122
Cardiopulmonary bypass time (min)	161 ± 78
X-Clamp time (min)	71 ± 38

**Table 2:** Postoperative mortality and complications

30-day mortality	170 (30.8 %)
30-day mortality in patients receiving IABP intraoperatively	159/512 (31.1 %)
30-day mortality in patients receiving IABP postoperatively	11/39 (28.2 %)
Duration of IABP-support (days)	5.6 ± 4.9
Ventilation time (days)	6.0 ± 10.4
ICU-stay (days)	9.5 ± 12.8
Norepinephrine >0.4 µg kg^−1^ min^−1^	134 (24.2 %)
Epinephrine >0.2 µg kg^−1^ min^−1^	397 (71.9 %)
Max. creatine kinase (mg mL^−1^)	2144 ± 3177
Max. CK-MB (mg mL^−1^)	141 ± 152
Hemodialysis	175 (31.7 %)
Rethoracotomy due to bleeding	58 (10.7 %)
Stroke or prolonged neurological deficit	55 (10.0 %)
Sepsis	76 (13.8 %)
Laparotomy	43 (7.8 %)

### Univariate and multivariate analyses for 30-day mortality

All the variables listed in Tables 1 and 2 were analyzed and following factors listed in Table 3 were identified as risk factors for 30-day mortality: gender, current smoker, age above 70 years, the combination of coronary artery bypass grafting and valve surgery, heart transplantation, operation time above 300 min, cardiopulmonary bypass time above 180 min, aortic X-clamp time above 120 min, use of hypothermic circulatory arrest, need of norepinephrine more than 0.4 µg kg^−1^ min^−1^, need of epinephrin more than 0.2 µg kg^−1^ min^−1^, re-thoracotomy due to bleeding, need for hemodialysis, stroke or prolonged neurological deficit, laparotomy, sepsis, mechanical ventilation exceeding 6 days and maximal creatinkinase above 3000 U/L. A multivariate analysis among those factors was performed and following factors as listed in Table 3 were identified as independent risk factors: gender, age above 70 years, the combination of coronary artery bypass grafting and valve surgery, aortic X-clamp time above 120 min, use of hypothermic circulatory arrest, need of norepinephrine more than 0.4 µg kg^−1^ min^−1^, need for hemodialysis and maximal creatinkinase above 3000 U L^−1^.

**Table 3:** Univariate and Multivariate analyses for 30-day mortality

	Mortality	Univariate analysis	Multivariate analysis			
		*p*	*p*	OR	95 % CI	
Gender						
Male	90/372 (24.2 %)	0.0001	0.001	2.211	1.359	3.598
Female	80/180 (44.4 %)					
Current smoker						
Yes	19/98 (19.4 %)	0.007	0.404	0.746	0.374	1.486
No	151/454 (33.3 %)					
Age >70 years						
Yes	107/266 (40.2 %)	0.0001	0.029	1.739	1.059	2.856
No	63/286 (24.2 %)					
CABG + valve						
Yes	56/118 (47.5 %)	0.0001	0.001	3.793	1.734	8.295
No	114/444 (26.3 %)					
Heart transplantation						
Yes	2/22 (9.1 %)	0.024	0.053	0.185	0.034	1.021
No	168/530 (31.7 %)					
Operation time >300 min						
Yes	96/247 (38.9 %)	0.0001	0.628	1.170	0.620	2.206
No	74/305 (24.3 %)					
CPB time >180 min						
Yes	73/173 (42.2 %)	0.0001	0.523	1.260	0.619	2.565
No	92/371 (44.4 %)					
Aortic X-clamp time >120 min						
Yes	24/46 (52.2 %)	0.001	0.021	2.817	1.171	6.776
No	141/497 (28.4 %)					
Use of hypothermic circulatory arrest						
Yes	18/30 (60.0 %)	0.0001	0.002	5.101	1.786	14.571
No	147/513 (28.7 %)					
Norepinephrine >0.4 µg kg^−1^ min^−1^						
Yes	56/134 (41.8 %)	0.0001	0.016	1.881	1.124	3.146
No	106/410 (25.9 %)					
Epinephrine > 0.2 µg kg^−1^ min^−1^						
Yes	130/397 (32.7 %)	0.013	0.370	1.283	0.744	2.215
No	32/147 (21.8 %)					
Rethoracotomy due to bleeding						
Yes	25/58 (43.1 %)	0.032	0.190	1.588	0.796	3.169
No	145/494 (29.4 %)					
Postoperative hemodialysis						
Yes	99/175 (56.6 %)	0.0001	0.0001	5.970	3.414	10.441
No	71/377 (18.8 %)					
Stroke or prolonged neurological deficit						
Yes	24/55 (43.6 %)	0.030	0.067	1.973	0.953	4.082
No	146/497 (29.4 %)					
Laparotomy						
Yes	22/43 (51.2 %)	0.003	0.553	1.282	0.564	2.913
No	148/499 (29.1 %)					
Sepsis						
Yes	38/76 (50.0 %)	0.0001	0.981	1.009	0.482	2.111
No	132/476 (27.7 %)					
Ventilation time ≥6 days						
Yes	67/145 (46.2 %)	0.0001	0.932	0.972	0.515	1.837
No	100/402 (24.9 %)					
Max. CK ≥ 3000 mg mL^−1^						
Yes	50/111 (45.0 %)	0.0001	0.05	1.714	1.002	2.973
No	120/441 (27.2 %)					

### Establishment of a scoring system

**Fig. 1:**
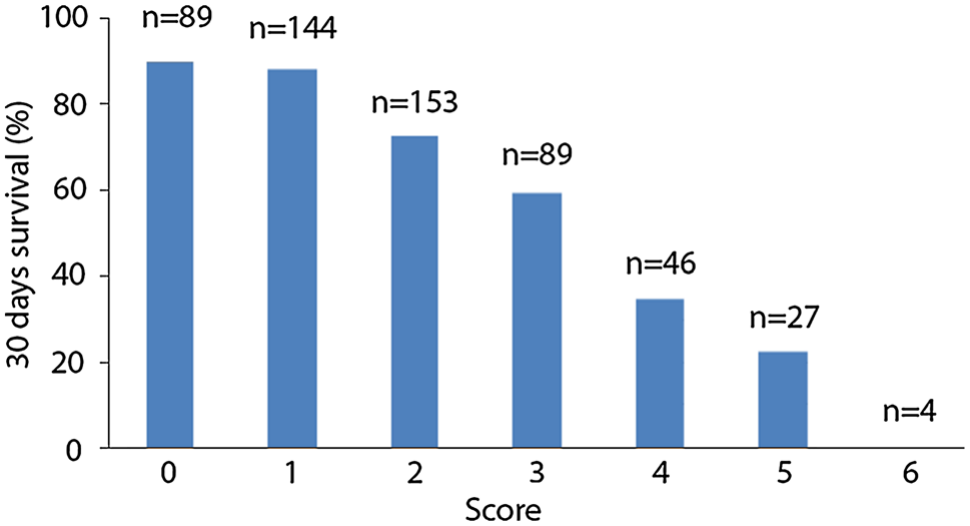
30 day mortality according to the scoring system

**Fig. 2:**
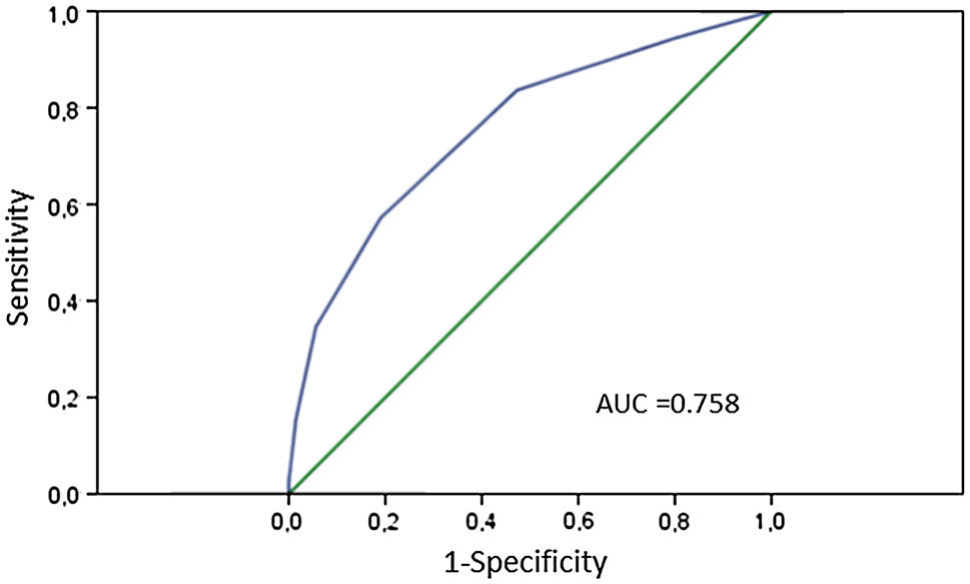
**a** Estimated cumulative survival curve for the entire patient cohort. **b** Estimated survival curve stratified for score values according to the scoring system

According to the result of the multivariate analysis, one scoring point was given for each independent risk factor; female (OR 2.211), age above 70 years (OR 1.739), the combination of coronary artery bypass grafting and valve surgery (OR 3.793), aortic X-clamp time above 120 min (OR 2.817), use of hypothermic circulatory arrest (OR 5.101), need of norepinephrine more than 0.4 µg kg^−1^ min^−1^ (OR 1.881), need for hemodialysis (OR 5.970) and maximal creatinkinase above 3000 U L^−1^ (OR 1.714). A summation of added points was calculated as the actual score. The distribution of patients and the observed 30-day mortality dependent on the score is shown in Fig. 1. The 30 days mortality was 10.1 % in patients with 0 point, 11.8 % in patients with 1 point, 27.5 % in patients with 2 points, 40.4 % in patients with 3 points, 65.2 % in patients with 4 points, 77.8 % in patients with points 5 and 100 % in patients with points 6. Discrimination by ROC analysis was 0.76 (0.71–0.80, Fig. 2).

### Long-term survival according to the scoring system

**Fig. 3:**
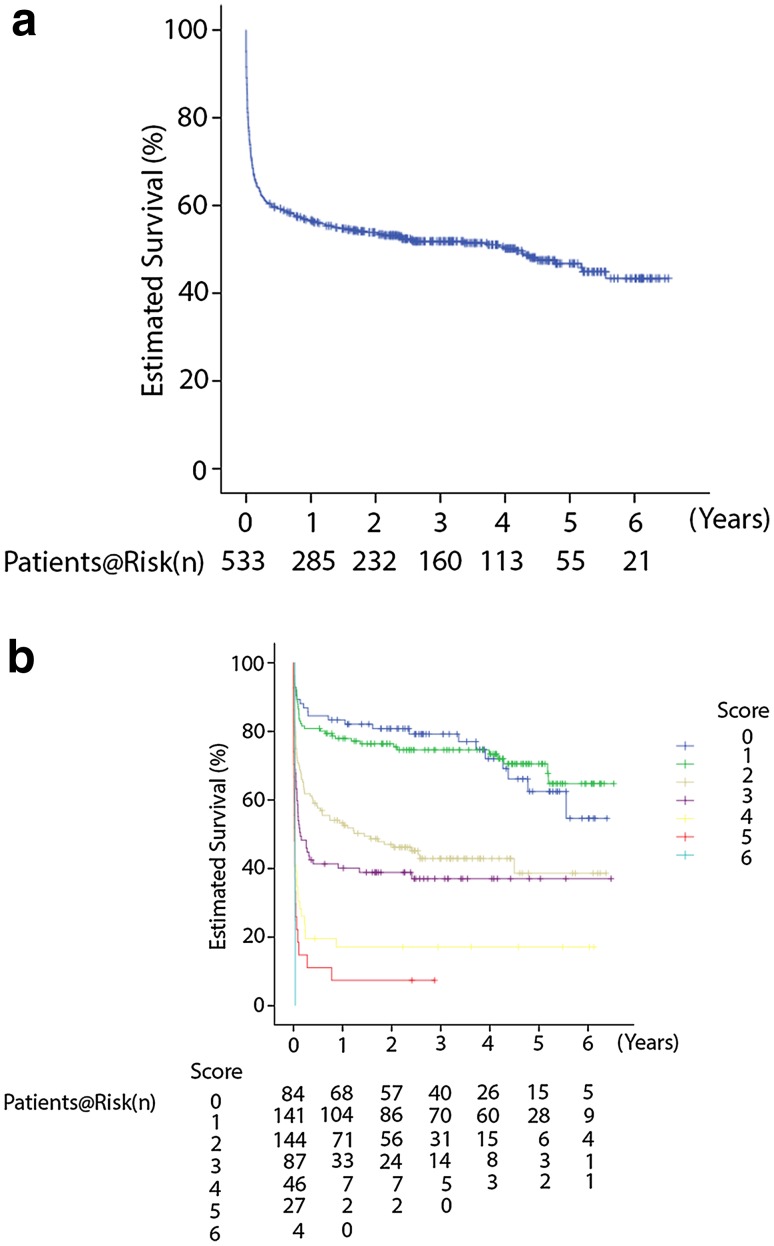
ROC curve for the scoring system

Survival curve in the entire patient cohort is shown in Fig. 3a. Overall cumulative survivals were 69.2 % after 30 days, 59.3 % after 180 days, 56.6 % after 1 year and 46.8 % after 5 years. Survival curve according to the scoring system is shown in Fig. 3b. Patients with 4 or more points had extreme poor long-term survival.

## Discussion

The crucial findings of the present study were: (1) significant risk factors could be identified in a relatively large patient cohort needing IABP after cardiac surgery, (2) the prediction of 30-day mortality was possible with our scoring system based on a multivariate analysis, and (3) long-term survival was extremely poor in patients with high score values.

In the present study, gender, age above 70 years, combined coronary and valve surgery, aortic X-clamp time above 120 min, use of hypothermic circulatory arrest, need of norepinephrine more than 0.4 µg kg^−1^ min^−1^, need for hemodialysis and maximal creatinkinase levels above 3000 U L^−1^ were independent risk factors for early mortality. Interpreting our findings, female and elderly patients, patients undergoing extensive surgery and displaying postoperative vasoplegia, perioperative myocardial infarction or postoperative renal failure were at particularly elevated risk for early mortality. These findings per se may not be surprising because all of these factors are already well known risk factors in cardiac surgery, independent of a need for IABP-support [8–10]. Nevertheless, our findings would be worth, on the one hand to confirm those risk factors in a large patient cohort, on the other hand to extend their focused validation to a cohort of critically ill patients needing IABP-support due to LOS after cardiac surgery.

With our new scoring system, a prediction of 30 days mortality was possible. Efforts to establish a scoring system designed for patients with an IABP-support due to LOS after cardiac surgery have already been made by others to know who would need a further therapy escalation with an ECMO or a VAD, as already depicted in the introduction. We consider that scoring systems based on early postoperative hemodynamic parameters as reported by two German large volume centers, the German Heart Center Berlin Group [4] and Bad Oeyenhausen Group [5], are particularly reliable scoring systems to detect such patients. One remarkable disadvantage of these two scoring systems is; however, that they are not designed to be applied on patients who are initially stable on a marginal level with an IABP-support and moderate or high dose of inotropic support. Notably, a prediction of the outcome in the course of postoperative ICU-stay, as it may become possible with our new scoring system, appears of significant clinical relevance.

To our best knowledge, there has been only one study reported by Arafa OE et al. in the year 1998 on long-term survival in patients needing an IABP-support due to LOS after cardiac surgery [2]. The authors analyzed long-term survival of 344 patients undergoing cardiac operations who required the perioperative use of an IABP from 1980 to 1989, and the survival rates were 40 % after 1 year and 32 % after 5 years. In contrast to their study, the survival rates in the present study were 57 % after 1 year and 47 % after 5 years, which partly may be explained by the advancement of cardiac surgical techniques and the intensive care therapy from the 1980’s to 2000’s. Nevertheless, our results suggest that patients needing an IABP-support due to LOS after cardiac surgery still belong to a high risk cohort. This becomes obvious in an almost dramatic way, when patients with scores 4 or more and their outcome are analyzed in the present study. From this aspect, a further therapy escalation with an ECMO or a VAD should be reconsidered, particularly in patients with high score values, where careful selection deserves great attention of therapy guiding medical team.

As already suggested by the previous two reports on IABP scores [4, 5], all patients in whom an IABP-support is not enough to maintain appropriate circulation could theoretically be considered as candidates for a further therapy escalation with an ECMO or a VAD. However, one should be aware that an ECMO and a VAD are no magical devices capable of saving all patients suffering from a post-cardiotomy cardiogenic shock. The Leipzig group reported in the year 2010 on 517 patients treated with an ECMO for refractory postcardiotomy cardiogenic shock [11]. In their report, weaning from ECMO was successful in 63 % of the patients and 25 % could be discharged. Cumulative survivals were 18 % after 6 months, 17 % after 1 year and 14 % after 5 years [11]. In contrary, the results for patients receiving a VAD appear to be more encouraging [12]. A study based on the data from the Society of Thoracic Surgeon’s National Cardiac Database, early mortality after VAD-implantation in patients with postcardiotomy shock has dramatically decreased over time, reaching mortality a rate of only 41 % between 2002 and 2004 [13]. However, almost half of these patients received a VAD for weaning from cardiopulmonary bypass and about 40 % of all operations were performed on an elective base in this study. Therefore, it may be possible that a large percentage of the involved cases were performed with a VAD stand-by, which may have led to those excellent results [13].

Undoubtedly, there are many patients with postcardiotomy cardiogenic shock in whom an IABP-support is not enough, as revealed by the high 30-day mortality in the present study reaching 31 %. For such patients, further therapy escalation with an ECMO or a VAD is the last hope. However, not all the patients could be saved with an ECMO and a VAD, and therefore patient selection is of paramount importance, not only because of medical reasons, but also because of limited health care resources. Moreover, there also exist many patients in whom further therapy escalation is actually contraindicated and yet they cannot be weaned from IABP while ICU-stay prolongs, in some cases reaching 10 days or more. In such a situation, the physician team is expected to make a difficult and serious decision. We believe that our new scoring system may prove helpful to make a decision in all directions, be it a therapy escalation employing an ECMO or a VAD, be it maintaining the actual therapy with an IABP or be it the cessation of the therapy in the most complex cases.

During the study period, an ECMO or a VAD were used very restrictively in patients having postcardiotomy LOS in our institute and it is the clear limitation of this study. Therefore, two previous German studies [4, 5] may be better for identifying patients requiring an ECMO or a VAD due to postcardiotomy LOS. Nevertheless, the focus of the present study was rather on identifying patients in whom the therapy cessation may be justified. For example, therapy escalation from IABP to an ECMO or a VAD may not be indicated in an 85 year old patients having very poor left ventricular function due to perioperative myocardial infarction. In this meaning, the present study would be worth despite of the clear study limitation.

In conclusion, patients needing an IABP-support due to LOS after cardiac surgery still belong to a high risk cohort. Prediction of 30-day mortality was possible with our scoring system based on multivariate analysis, and patients with score values of 4 or more had obviously worse early and late survival. We believe that our new scoring system may be helpful to meet a decision in all directions; therapy escalation with an ECMO or a VAD, maintaining the actual therapy with an IABP or therapy cessation.
